# Supplementary material for: Comparative multiomics analysis of cell physiological state after culture in a basket bioreactor
Source: Sci Rep. 2022 Nov 23;12:20161. doi: 10.1038/s41598-022-24687-4 (PMC9686226; doi:10.1038/s41598-022-24687-4)
Supplement: Supplementary file 1 — Supplementary Information 1. [file 41598_2022_24687_MOESM1_ESM.zip › raw data/Transcriptomic raw data/7.AS/2.dasplot/TE1vsTC1/A3SS/8_USP24_NW_023666033.1_39512561_39512723_-@NW_023666033.1_39512561_39512726_-@NW_023666033.1_39549488_39551237_-.pdf]

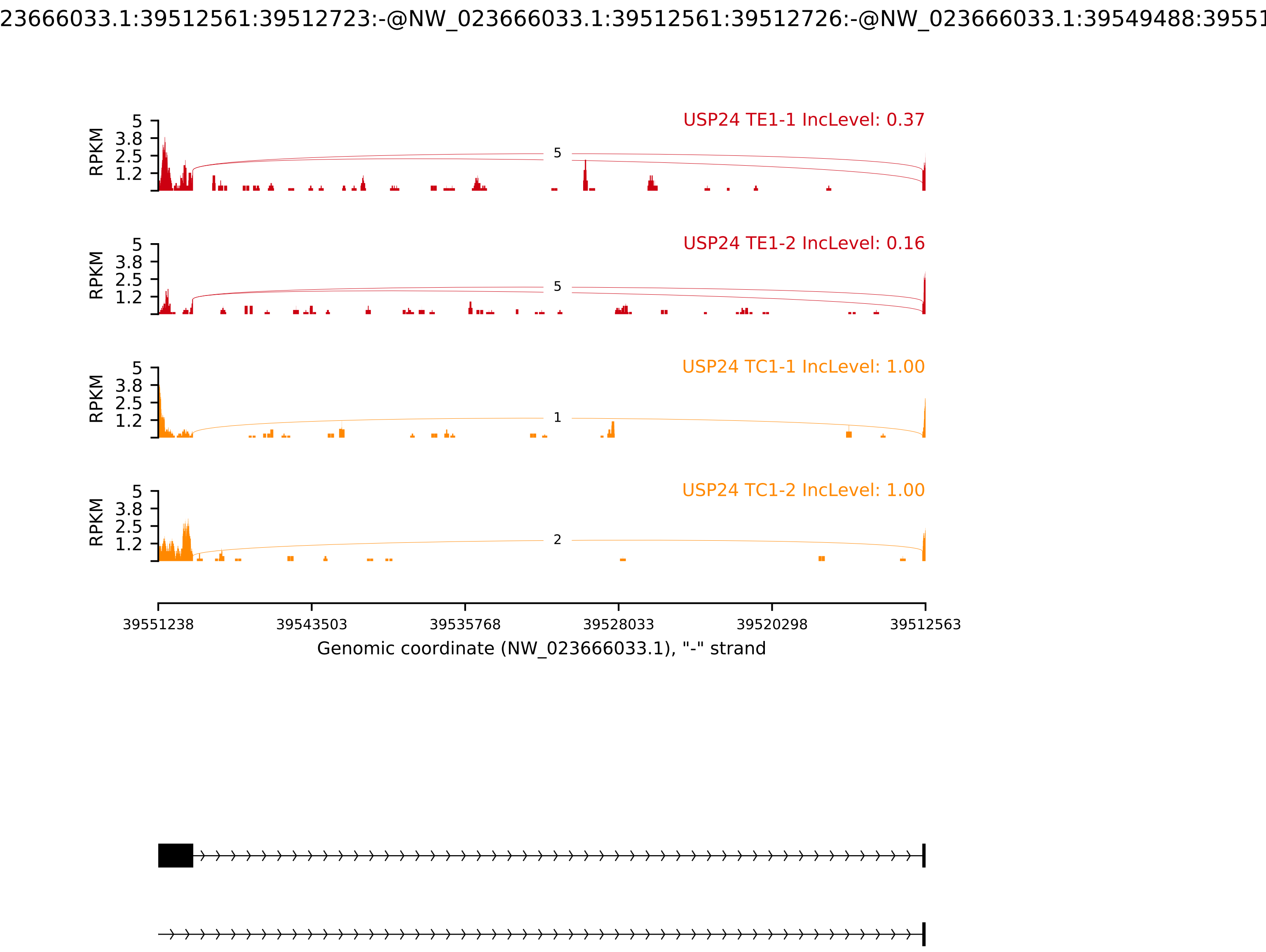

23666033.1:39512561:39512723:-@NW_023666033.1:39512561:39512726:-@NW_023666033.1:39549488:39551
RPKM
5
3.8
2.5
1.2
USP24 TE1-1 IncLevel: 0.37
5
USP24 TE1-2 IncLevel: 0.16
5
USP24 TC1-1 IncLevel: 1.00
1
USP24 TC1-2 IncLevel: 1.00
2
39551238
39543503
39535768
39528033
39520298
39512563
Genomic coordinate (NW_023666033.1), "-" strand
